# Former foodstuffs in feed: a minireview of recent findings

**DOI:** 10.1007/s11356-024-32695-2

**Published:** 2024-03-04

**Authors:** Karthika Srikanthithasan, Andrea Giorgino, Edoardo Fiorilla, Laura Ozella, Marta Gariglio, Achille Schiavone, Andrés Luis Martínez Marín, Elena Diaz Vicuna, Claudio Forte

**Affiliations:** 1 Department of Veterinary Sciences, University of Turin, 10095 Grugliasco, Italy; 2 R&D, technical and commercial manager, Dalma Mangimi Spa, 12030 Marene, Italy; 3 Departamento de Producción Animal, Universidad de Córdoba, 14071 Córdoba, Spain

**Keywords:** Former foodstuffs, Feed-Food competition, Livestock feed, Circular economy, Circular feed, Livestock performance

## Abstract

The sustainability of all productive activities, including livestock farming, becomes a fundamental challenge in the current scenario. Livestock production faces both old and new challenges related to climate change, food safety, and feed-food competition. The latter aspect has recently become a hot topic, and many researchers are turning their attention to this issue. According to circular economy principles, former foodstuffs have characteristics that make them a promising source of raw material for animal feed. The main objective of the present review is to provide a brief overview of the most recent studies (published between 2016 and 2022) addressing the dietary inclusion of former foodstuffs for livestock. The articles analyzed cover key findings from both in vitro and in vivo studies of former foodstuffs included in the diets for pigs, cows, and broilers, and assess the associated safety aspects. The articles provide information on livestock performances and product quality, as well as feed digestibility, fecal microbiota, and blood analysis. Although the evidence supports the inclusion of former foodstuffs in livestock diets as a safe, effective, and sustainable ingredient, this analysis of the most recent literature also highlights gaps in our knowledge that need to be filled. The present overview will help researchers plan future research and standardize and promote the inclusion of former food products in livestock diets.

## Introduction

FAO estimates that by 2050 the output of overall food production will need to increase by 70% in order to meet the nutritional requirements of the world population (FAO 2009). Other important drivers for future developments include changings in global supply chains, addressing societal concerns and ensuring a sustainable use of resources (Maria Binder 2019).

The livestock sector is currently dealing with numerous challenges, including food security, environmental emissions, climate change, and “feed-food competition” (Muscat et al. 2020). The latter term refers to “the tensions and trade-offs between two alternative uses for edible crops: direct consumption by humans versus feeding livestock” (Breewood and Garnett 2020).

The level of sustainable feed production needs to be increased and novel resources must be identified and exploited in accordance to the circular economy principles (FAO 2017). Europe is presently characterized by disruptions in supply chains due to the recent COVID-19 pandemic, wars, and fluctuations in the international market economy (Eurostat 2021). A recent publication by the European Feed Manufacturers' Federation (FEFAC 2022) sustains that the circular economy framework should reinforce the EU’s feed autonomy and defines a circular feed as a “non-food-grade ingredient recovered as a secondary raw material from the (local) circular economy with a low land-use footprint.”

Insect meal (Pinotti et al. 2019), co-products (Mackenzie et al. 2016), food leftovers (Pinotti et al. 2021), by-products (Karlsson et al. 2018), bakery by-products (BBP) (Humer et al. 2018), and former foodstuffs (FFs) (Pinotti et al. 2019) are a few of the possible sources of innovative raw materials being considered for livestock feed. Of the proposed solutions, studies based on the use of FFs in animal feed constitute research emerging from the principle of innovative circular economy (Breewood and Garnett 2020). According to the catalogue of feed materials (European commission 2022), FFs are “foodstuffs, […] manufactured for human consumption in full compliance with the EU food law, but which are no longer intended for human consumption […] and which do not present any health risks when used as feed.” Likewise, FFs processors play an important role in connecting the food and feed industries (EFFPA 2019a).

The objective of the present review was to provide a concise overview of the most recent studies on FFs inclusion in livestock feed, including studies addressing the effects on growth performances, nutrient digestibility, gut microbiota, hematological parameters, product quality focusing on both monogastrics and ruminants, particularly in poultry, and animal-derived food quality (Afzalzadeh et al. 2007; Almeida et al. 2011; França et al. 2012). Although few articles have covered this topic before, the existing literature lacks sufficient focus on poultry nutrition (Pinotti et al. 2021, 2023). This analysis also revealed the gaps in our knowledge which need to be filled for the feed industry to evolve and promote the use of FFs in animal nutrition.

## Methods

The keywords selected for the literature search were: “ex food,” “former foodstuffs,” “food leftovers,” “former food products,” “bakery products,” “bakery by-products,” and “bakery meal.”

The above keywords were used to search the Scopus, PubMed, and Google Scholar databases, and a total of 37 articles were identified. The selection of articles for inclusion followed specific criteria, involving a thorough review of full paper. Articles had to meet the following inclusion criteria: i) be published between January 2016 and October 2022; ii) clearly state the study type (safety, in vivo, or in vitro); iii) include the chemical composition of the diet being studied in both in vivo and in vitro studies. Articles not meeting one or more of the above criteria were excluded (*n*=19 out of 37 articles).

## Safety aspects

One of the reasons limiting the use of FFs is the perceived safety aspect. The literature on this topic mainly focuses on microbiological safety and the presence of packaging residues. Although FFs can generally be considered microbiologically safe for feed production due to their pre-thermal processing and nutritional quality (in terms of starch and fat content), having originally been destined for human consumption (Amato et al. 2017; Tretola et al. 2017b, 2019b). Another major concern is that packaging residues can lead to potential feed contamination. Although the level of contamination has been reported as negligible, several approaches are being explored to ensure a better risk assessment of the presence of packaging residues in FFs. Luciano et al. (2022b) highlighted the key techniques for detecting the different Packaging Remnants.

To meet our inclusion criteria for the literature in this mini-review, we decided to focus our analysis solely on experimental studies and exclude review articles from further discussion.

All the FFs analyzed in Tretola et al. (2017a) were reported to be microbiologically safe: total viable counts were within acceptable limits, and *Salmonella* spp. was absent. All the biological hazards tested (*Enterobacteriaceae*, *E. coli, Staphylococci, B. cereus, Clostridia,* yeasts, and molds) were below the thresholds set by Commission Regulation (EU) No. 142/2011 (European commission 2011).

Although packaging materials are strictly regulated in the EU (European commission 2009), their presence in FFs could limit their use in animal feed. Innovative methods have been developed to detect traces of plastic, aluminum foil, and cardboard in FFs.

Amato et al. (2017) applied the fast and sensitive gravimetric method based on the RIKILT technique for routine official controls, while Tretola et al. (2017a) showed that remote sample image analysis, involving computer vision technology coupled with a stereomicroscope, could be used to rapidly identify packaging remnants mainly aluminum particles in FFs. The results depend on the ability of the inspector to correctly recognize and quantify the different remnants. Both studies proved that the traces of packaging materials detected lay within the limits set by EC Regulation (European commission 2009).

In a different study, Tretola et al. (2019b) confirmed the capability of the Electronic Nose (E-Nose; a recently developed, rapid, and objective method) to distinguish between samples (administered as: “cleaned,” “as received,” and “spiked”). However, the E-Nose was not effective in analyzing mixtures of different FFs. This limitation depends on the complexity of the matrix, but the E-Nose was proven to be useful as a supporting tool able to facilitate stereomicroscope analysis, thus reducing the labor time and increasing objectivity.

Calvini et al. (2020) demonstrated the potential of multivariate image analysis for detecting packaging remnants using red, green, and blue channel microscope images of FFs acquired by a stereomicroscope equipped with a digital camera to do a fast, non-invasive, and inexpensive analysis. The authors’ preliminary tests performed on six different commercial samples of FFs by two different approaches such as pixel-level analysis and image-level analysis led to results, especially when the residues were of colors different from that of the matrix.

The findings from the above-cited results confirmed the high quality of the FFs analyzed and the negligibility of their microbiological risk. Therefore, further research and advancement of specific models for different types of FFs would be needed to assess the safety aspects of FFs in more depth, considering all the possible limiting factors of dealing with a non-standardized matrix derived from different production chains, each characterized by different risks. It should be noted that European law requires that all products from the food sector destined to feed production are, by definition, neither harmful nor dangerous (EFFPA 2019b).

## In vitro studies

A list of the analyzed publications exploring FFs in livestock diets using in vitro tests is reported in Table 1.

**Table 1:** In vitro studies

Reference	Species	Products used	Number of tested products	Analysis	Results
Giromini et al. (2017)	Pig	FFs	6	Chemical analysis (% DM)	CP: 10.0; Starch: 52.4; EE: 10.1; NFE: 61.2-74.7; ash: 2.3; NSC: 58.5-79.3
				Energy values (MJ kg^–1)^	DE: 17.2; ME: 16.9
				Digestibility (%)	IVD: 88.2
Ottoboni et al. (2019)	Pig	FFs	6	Chemical analysis (g kg^-1^ DM)	Feed CTR: Moisture: 91 g kg^-1^, CP: 208, EE: 59, NDF: 126, Sugars: 72, Starch: 362, Ash: 56 Feed FFs30%: Moisture: 97 g kg^-1^, CP 206, EE: 59, NDF: 91, Sugars: 97, Starch: 426, Ash: 49
				Carbohydrate digestion	HI: 104.5; pGI: 105.9 C∞ range: 62.32-99.75/100g total carbohydrates k values: 0.05-0.21/min
Liu et al. (2018)	Pig	BM	46	Chemical Analysis (% DM)	DM: 91.84; CP: 12.20, AEE: 9.38, Starch: 44.61; NDF: 13.77, ADF: 6.18, Lysine: 0.35, Methionine: 0.19, Threonine: 0.38, Tryptophan: 0.13
				DM digestibility (%)	IVDMD: 79.06
				Energy digestibility (%)	IVGED: 74.84

FFs former foodstuffs, BM bakery meal, DM dry matter, CP crude protein, EE ether extract, NFE nitrogen free extracts, NSC non-structural carbohydrates, IVD in vitro digestibility, DE digestible energy, ME-metabolizable energy, NDF neutral detergent fiber, HI hydrolysis index, pGI predicted glycemic index, (C∞) potential digestibility of carbohydrates, k digestion rate/ min, AEE acid hydrolyzed ether extract, ADF acid detergent fiber, IVDMD in vitro dry matter digestibility, IVGED in vitro energy digestibility

Leftovers from the confectionery industry, defined as a “fortified version of cereals” (Giromini et al. 2017), are one form of FFs. They offer a valuable energy source characterized by a high sugar/starch content, and in some cases, they may also be fat enriched (such as cookies, wafers, and chocolate). BBP, on the other hand, as in the case of bakery meal (BM), are also characterized by a high carbohydrate content, and are mainly made of a mixture of bread, breakfast cereal, biscuits, and similar.

The chemical composition of FFs can represent a limiting factor for their use in livestock feed, with special emphasis on starch and fat contents and their digestibility. The passage of this kind of products through an intermediate processor is needed to ensure consistency among batches and to provide a high-quality feed (Pinotti et al. 2021).

Ottoboni et al. (2019) evaluated the predicted glycemic index (pGI) and hydrolysis index (HI) in six samples of FFs (bakery and confectionary ex-food) and two pig compound feeds (commercial feed) as control (Feed CTR), and CTR enriched with 30% of FFs (Feed FF30%). Corn meal, heat processed wheat also were included as control feed ingredients. The HI and pGI of all the FF samples were higher than those of unprocessed corn. This was related to the nature and the processing of the FFs, which were also characterized by high variability among the different samples. Regarding the two pig feeds, no differences in either the HI or GI were observed between the Feed CTR and FF30%. The authors also assessed total carbohydrate digestion during seven different incubation times (from 0 to 180 min) as a measure of digestion kinetics. No difference was found at 0 min (C_0_) and in the potential digestibility of carbohydrates (C∞). All FFs samples showed higher cumulative in vitro glucose release compared with unprocessed maize and heat processed wheat samples. The authors reported that all the FFs were derived from cooked ex-foods in which the starch was heat treated. It should be borne in mind that the cooking, processing, and the fat content of these ex-foods may have affected the in vitro glycemic responses recorded.

The study by Giromini et al. (2017) tested six samples of mixed FFs and compared them with wheat as control. The data are reported in Table 1. The authors stated that the high nitrogen free extracts, non-structural carbohydrates, starch, and fat concentration indicated FFs as valuable energy sources. The digestible energy (DE) and metabolizable energy (ME) values confirmed their characterization as energy-dense feed ingredients. The average in vitro digestibility (IVD) value of the FFs studied (88.2%) was comparable to that of wheat (90.6%).

A study by Liu et al. (2018) evaluated a total of 46 different samples of bakery meal (BM) sourced from different regions of the USA. Comparing these samples according to their geographic origin revealed only small differences in their chemical composition and no differences in their digestibility. The average concentrations are summarized in Table 1. As stated by the author, the results show that BM can be considered a substitute for corn, providing not only energy and starch, but also minerals. Ingredients with high concentrations of phosphorus and phytate (bran and canola co-products) are often included in BM. This explains why the BM used in Liu et al. (2018) contained more than 40% starch, and the concentrations of acid detergent fiber (ADF), neutral detergent fiber (NDF), and phytate-bound phosphorus were greater than in cereal grains.

The results of the in vitro studies examined here show that both similarities and differences exist between the properties of FFs and BBP depending on their composition (i.e., the ingredients used) and the geographic region of origin. Intermediate processors, who are able to buy FFs from Food manufacturers and produce feed ingredients, are needed to ensure qualitative consistency in the nutritional traits of FFs and BBP (Pinotti et al. 2021).

## In vivo studies

A summary of the in vivo studies conducted since 2016 on FFs in livestock diets is reported in Table 2. The main parameters to consider when testing alternative ingredients for inclusion into animal diets are nutritional content, palatability, and digestibility. Kaltenegger et al. (2020, 2021) reported that the inclusion of BBP (15% or 30%) into the diets of 24 lactating Simmental cows resulted in an increase in total dry matter intake compared with the control diet; digestible organic matter also increased linearly with increasing levels of BBP inclusion. The data obtained from analyzing the fecal microbiota highlighted an increased risk of dysbiosis due to diversity loss characterized by an increased amount of amylolytic bacteria and a decreased number of cellulolytic populations. These results were in accordance with previous in vitro studies, conducted by the same team (Humer et al. 2018), in which the BBP inclusion was responsible for a decrease in the diversity of the ruminal microbial population and reduced fiber degradation. The results of the paper led the authors to state the need for further studies designed to consider “-omics” techniques in order to better define the effects of BBP inclusion in dairy cows’ diet.

**Table 2:** In vivo studies

Reference	Species	Products	Inclusion (% in DM basis)	Analysis	Results
Humer et al. (2018)	Cows – Holstein, nonlactating rumen-cannulated (n=3)	BBP	0, 15, 30, 45	Performance	-
				Digestibility and Energy	-
				Gut and Microbiota	Data reported for 0, 15, 30, 45% BBP Decrease of the pH of incubation fluid: 6.63, 6.60, 6.60, 6.59 Comparable total SCFA concentration (mM) Decreased microbial diversity and fiber degradability for 45% BBP
				Blood Analysis	-
				Product quality	-
Kaltenegger et al. (2020)	Cows- Simmental, mid-lactating (n=24)	BBP	0, 15, 30	Performance	Data reported for 0, 15, 30% BBP DMI (kg/d): 23.0, 24.7, 24.9 Increased peNDF intake (min/kg)-eating: 50.7, 56.0, 70.1 Increase Milk yield (kg/d): 30.6, 32.1, 35.1 ECM (kg/d): 29.4, 31.5, 34.3
				Digestibility and Energy	-
				Gut and Microbiota	Data reported for 0, 15, 30% BBP Calculated SARA index of pH 5.8: 5.88, 3.23, 4.02 SARA index (min pH<5.8/kg of DMI): 27.6, 16.8, 22.5
				Blood Analysis	Data reported for 0, 15, 30% BBP Increased cholesterol (mg/dL): 180, 214, 257. Increased BHBA: (mmol/L): 0.054, 0.132, 0.119 Increased NEFA: 0.024, 0.038, 0.052
				Product quality	Data reported for 0, 15, 30% BBP Increased milk fat content (%): 3.59, 3.75, 3.90 Decreased milk protein (%): 3.63, 3.64, 3.51 Decreased MUN (mg/dL) concentration: 27.1, 23.8, 20.2
Kaltenegger et al. (2021)	Cows- Simmental, mid-lactating (n=24)	BBP	0, 15, 30	Performance	Data reported for 0, 15, 30% BBP Increased DOMI related to the BW ^0.75^ (g/kg): 104, 109, 123
				Digestibility and Energy	Data reported for 0, 15, 30% BBP Increased ATTD (%) of DM: 68.6, 70.1, 75.1; OM: 70.3, 71.8, 76.4; EE: 60.3, 64.0, 75.1; NDF: 51.6, 54.9, 64.2; Ash: 46.7, 48.3, 58.7
				Gut and Microbiota	Data reported for 0, 15, 30% BBP Increased fecal VFA concentration (μmol/g): 74.6, 79.1, 83.1 Reduced fecal pH: 6.61, 6.60, 6.47 Declined richness and diversity indices of the fecal microbiota
				Blood Analysis	-
				Product quality	-
Tretola et al. (2019c)	Piglets- Large White*Landrace, Post weaning (n=12)	FFs	0, 30	Performance	Data reported for 0, 30% FFs Lower FCR (kg/kg): 1.55, 1.39
				Digestibility and Energy	Data reported for 0, 30% FFs Increased IVD (%): 83.8, 86.5 Increased ATTD (%): 78.60, 83.30
				Gut and Microbiota	-
				Blood Analysis	Data reported for 0, 30% FFs (day16) Increased Glucose (mmol/L): 5.08, 6.18 Reduced Urea (mmol/L): 1.58, 1.03
				Product quality	-
Tretola et al. (2019a)	Piglets- Large White*Landrace, Post weaning (n=12)	FFs	0, 30	Performance	-
				Digestibility and Energy	-
				Gut and Microbiota	Decreased bacterial abundance, diversity, and stability in large intestine (FFs diet)
				Blood Analysis	-
				Product quality	-
Luciano et al. (2021)	Piglets-- Large White*Landrace, post-weaning (n=36)	FFs-C FFs-B	0, 30, 30	Performance	No effect on growth performance (35–42 days)
				Digestibility and Energy	Data reported for 0, 30% FFs-C, 30% FFs-B, respectively. ATTD of DM (g/100 g DM) (35–42 days): 89.4, 86.1, 90.3
				Gut and Microbiota	-
				Blood Analysis	-
				Product quality	-
Luciano et al. (2022a)	Experiment 1 Pigs, weanling barrows (n=80)	BM1 + Phytase and BM2 + Phytase (Phytase levels: 0, 500, 1000, 1500, 3000 FTU/kg)	98.49	Performance	-
				Digestibility and Energy	P intake (g/d) at 0 FTU/kg: BM1-1.21; BM2- 2.05 P absorption (g/d) with 3000 FTU/kg of phytase: BM1- 0.96; BM2 -1.46 ATTD of P with 3000 FTU/kg of phytase: BM1- 0.746; BM2- 0.696 STTD of P with 3000 FTU/kg of phytase: BM1- 0.851; BM2 -0.756
				Gut and Microbiota	-
				Blood Analysis	-
				Product quality	-
	Experiment 2 Pigs, Newly Weaned (n=160)	BM	Phase 1 (1-14days) 0, 12.9, 25.8, 38.5, 52.3 Phase 2 (15- 35days) 0, 13.5, 27.1, 40.8, 54.4	Performance	Data reported for 0, 13.5, 27.1, 40.8, 54.4 BM, respectively Phase 2: Decrease the gain to feed ratio : 0.70, 0.70, 0.63, 0.63, 0.53
				Digestibility and Energy	-
				Gut and Microbiota	Not affect fecal scores
				Blood Analysis	No effects on BUN, total protein, and albumin concentrations
				Product quality	-
Tretola et al. (2022)	Piglets- Large White*Landrace, Post weaning (n=36)	FFs-C FFs-B	0, 30, 30	Performance	-
				Digestibility and Energy	-
				Gut and Microbiota	No differences in the alpha diversity indexes, abundance, and biodiversity indexes of the microbial community No effect on fecal concentration of VFAs
				Blood Analysis	-
				Product quality	-
Casas et al. (2018)	Pigs, young growing cannulated (n=22)	BM	93.8	Performance	-
				Digestibility and Energy	Lower AID (45.3%) and SID (65%) of CP
				Gut and Microbiota	-
				Blood Analysis	-
				Product quality	-
Zhang and Adeola (2017)	Broiler-Ross 708 (n=320)	BM	0, 20, 40	Performance	No effect on growth performance
				Digestibility and Energy	Data reported for 0, 20, 40 BM, respectively. IDE (kcal/kg DM): 3.963, 3.832, 3.862
				Gut health and Microbiota ecosystem	-
				Blood Analysis	-
				Product quality	-
Stefanello et al. (2016)	Broiler-Ross 708 (n=780)	BM	0, 10, 20	Performance	-
				Digestibility and Energy	Data reported for 0, 10, 20 BM, respectively. Ileal digestibility coefficients; decreased DM: 0.692, 0.681, 0.675; Nitrogen: 0.767, 0.738, 0.686; Energy: 0.737, 0.725, 0.713; IDE (kcal/kg of DM): 3496, 3412, 3271 Decreased in retention of DM: 0.678, 0.669, 0.664, Nitrogen; 0.602, 0.582, 0.523, Energy: 0.730, 0.720, 0.705
				Gut and Microbiota	-
				Blood Analysis	-
				Product quality	-

BBP bakery by products, FFs former foodstuffs, (FFs-C) sugary confectionery products, (FFs-B) salty bakery products, BM bakery meal, DM dry matter, SCFA short-chain fatty acids, DMI dry matter intake, peNDF physically effective nitrogen detergent fiber, ECM energy-corrected milk, SARA subacute ruminal acidosis, BHBA β-hydroxybutyrate, NEFA non-esterified-fatty acids, MUN milk urea nitrogen, DOMI digestible organic matter intake, ATTD apparent total-tract digestibility, OM organic matter, EE ether extract, NDF neutral detergent fiber, FCR feed conversion ratio, IVD in vitro digestibility, FTU phytase units, STTD standardized total tract digestible, P phosphorus, BUN blood urea nitrogen, VFA volatile fatty acids, AID apparent ileal digestibility, SID standardized ileal digestibility, IDE ileal digestible energy

The same studies also reported a linear increase in daily milk yield and energy-corrected milk. Milk composition was also modified: the fat to protein ratio increased as did the concentration of milk urea nitrogen (27.1 mg/dL in controls vs 20.2 mg/dL in cows fed 30% BBP).

BBP inclusion had a marked impact on the blood metabolic profile of cows. The diurnal concentration of glucose and plasma insulin linearly declined by feeding BBP and decreased throughout the day. Although non esterified fatty acids (NEFA) and beta-hydroxybutyrate (BHB) remained at low levels (NEFA <0.05 mmol/L; BHB <0.6 mmol/L), their concentrations increased linearly with increasing level of BBP in the diet (Kaltenegger et al. 2020). Furthermore, the apparent total tract digestibility (ATTD) of other parameters listed in Table 2 also increased as the BBP level in the diet increased (Kaltenegger et al. 2021).

FFs and BBP in the diet can affect monogastric performance in several aspects. In a study conducted by Tretola et al. (2022), 28-day-old postweaning piglets (*n*=36) were divided into three groups, each receiving a different diet: a conventional diet (control), a diet in which cereals were partially replaced (30% w/w) by sugary confectionery products (FFs-C), and one in which they were replaced by salty BBP (FFs-B). The study demonstrated that the inclusion of FFs can contribute to obtaining balanced diets with a chemical composition similar to that of conventional ones. Neither the FFs-C nor FFs-B diets were found to affect the abundance or biodiversity indexes of the microbial community. In fact, a few taxa normally attributed to a healthy gut actually increased compared to the control. The experimental diets had no impact on the production of volatile fatty acids as detected in the feces.

Luciano et al. (2022a) performed two experiments which involved the addition of BM with or without phytase in the diet for pigs. The first experiment compared two diets based on two distinct sources of BM (mainly different for protein - 96.1 g/kg versus 152.6 g/kg - and starch 451.0 g/kg versus 382 g/kg - content identified as BM1 and BM2 respectively) supplemented with microbial phytase (0, 500, 1000, 1500, 3000 FTU/kg). The diets obtained were administered to weanling barrows (*n*=80). BM2 resulted in greater phosphorus intake compared with BM1. Phosphorus absorption in pigs fed the BM2 diet supplemented with 3000FTU/kg of phytase was higher than that in pigs fed the BM1 diet. Indeed, increases in both the ATTD of phosphorus and the standardized total tract digestible (STTD) of phosphorus were observed in BM1 compared with BM2. The second experiment showed that using BM to replace corn meal at different proportions (0, 250, 500, 750, or 1000 g/kg) in newly weaned pigs (*n*=160) can decrease the gain to feed ratio (G: F) without modifying the pigs’ final body weight.

In the study performed by Luciano et al. (2021), standard ingredients were replaced by bakery (FFs-B: 30%) or confectionary (FFs-C: 30%) FFs in 36 post-weaning piglets. The authors found no differences in growth performance between the groups. Likewise, in the reports by Tretola et al. (2019c, a), in which the feed given to post-weaning piglets (*n*=12) was partially replaced (30%) with FFs, no differences were found in final body weight, but the piglets fed FFs had a lower feed conversion rate compared with those receiving the control diet. In the same study the IVD values for the control diet were lower than those for the FFs diet, and the final ATTD was higher in piglets fed FFs (83.30% in FFs vs 78.60% in control). Luciano et al. (2021) recorded a similar ATTD of dry matter from piglets fed FFs-B and control group, while a decrease was recorded in piglets fed FFs-C. The analysis of Casas et al. (2018), who compared the digestibility of BM with other sustainable raw materials in 22 young growing pigs, showed a lower level of standardized ileal digestibility (%) of both crude protein and amino acids.

The studies by Luciano et al. (2021, 2022a) found no significant differences in the hematological parameters between the dietary groups over the course of the entire experiment, whereas Tretola et al. (2019c) reported changes in blood metabolism when administering FFs to piglets, recording an increased glucose and a decreased urea concentration.

In Tretola et al. (2019a), the microbiota in 12 piglets fed the same diets as reported in Tretola et al. (2019c) were analysed. The authors reported a decrease in bacterial diversity in pigs receiving the FFs diet compared with control, with minor differences in taxa definition, consistent with the results reported by Kaltenegger et al. (2021) for dairy cows. Considering that the use of BBP had no detrimental effects on in vivo performance, further studies are needed to understand in greater depth how the microbiota changes when BBP or other FFs are included in the diet.

Products from the food industry have also been evaluated for their inclusion in poultry diets. In the study presented by Zhang and Adeola (2017), a corn-soybean meal was partly replaced with 200 g BM/kg and 400 g BM/kg for Ross 708 broiler chickens (*n*=320). The inclusion of BM did not affect the birds’ growth. Adding BM linearly decreased ileal digestible energy (IDE) compared with canola meal and cottonseed meal, but had no effects on DM and energy metabolizable coefficients, metabolizable energy, or the nitrogen correlated metabolizable energy values.

In Stefanello et al. (2016), Ross-708 chicks (*n*=780) were fed a diet in which BM was included at 0 g/kg (control), 100 g/kg (L-BM), and 200 g/kg (H-BM), replacing the same weight of cereals. The ileal digestibility coefficients of DM and IDE decreased linearly according to increased levels of dietary BM inclusion. A linear decrease in DM, N, and energy retention was also observed, as was a quadratic response in N retention and ME.

All of the reported studies support the inclusion of FFs in feed for ruminants and monogastric animals. Additionally, in vivo the in vivo findings align with those observed in in vitro studies. The presented in in vivo studies predominantly focus on dairy cattle, pigs and poultry. In overall, a comprehensive investigation of meat quality and safety in both monogastrics and ruminants are lacking, particularly regarding carcass traits, and sensory analysis for sustainable meat production. Notably, only two cited articles focus solely broilers, providing data on growth performance and digestibility. This highlights the need for more detailed and in-depth research to hold the species-specific nutritional requirements, production impacts, digestive physiology, health status, and consumer safety concerning product quality when utilizing an alternative ingredient as FFs.

## Conclusion and future perspective

This review summarizes the recent findings from the literature on the characteristics of FFs as feed ingredient in modern livestock system. The reported data demonstrates the possibilities of utilizing FFs in animal nutrition in align with circular economy principles, evidently meeting safety standards outlined in EU feed safety regulations about FFs. Nevertheless, further studies focusing on safety aspects are necessary. However, the review also highlights how the involvement of FFs in strategies aimed at increasing the sustainability of livestock feed is not being fully exploited at present. One reason for this arises from the lack of information on the effects of a diet containing a high percentage of FFs on animal productivity, gut ecosystems, product quality and nutritional composition together with an assessment of the animal welfare and sustainability features. Most of the examined studies focused on dairy cows and pigs, whereas the inclusion of FFs in poultry diet and their knock-on effects on production have yet to be properly defined.

Chicken meat is cost-effective and widely available source of protein source, making poultry production a significant component of the meat industry, driven by increasing global demand. However, the sustainability of chicken production and consumption remains a subject of debate. As a result, ongoing efforts are directed toward enhancing the environmental sustainability and economic viability in chicken meat production. The feed industry is experiencing a crucial development phase, incorporating alternative feed ingredients. One of the sustainable feeding strategies involves partially replacing the conventional ingredients, such as soybean and corn meal, with FFs in balanced diet formulations. Assessing the sustainability and efficiency of incorporating FFs into poultry diet requires a broad aspect such as feed efficiency, animal productivity and health, and production costs. This approach aims to establish excellence in both environmental and economic aspects of poultry farming.

Further multidisciplinary in vivo studies are needed to validate the possible inclusion of FFs in the diet of different species such as beef cattle, goat, lamb, laying hens, ducks at different inclusion levels. This aims to identify the maximum or optimum level of FFs inclusion for each specific-species’ nutritional requirements. Since, FFs are already available in the market, it is important to determine the consumers’ perception regarding their use in animal nutrition and the final products. In this context, investigating FFs’ effect on meat quality and sensory attributes, and egg production are essential. This assessment will aid to uncover any misconceptions and facilitate the education of farmers and consumers about the availability and potential use of FFs towards sustainability and circular economy. Such insights would allow to more awareness and positive image of FFs, eventually increasing their acceptability. Furthermore, a life cycle cost analysis should also be performed to clarify whether the conversion of FFs is cost-effective for both the farmers and the processing industries involved.

Despite these challenges, there are many opportunities for using FFs in livestock feed. As the world's population continues to grow and the demand for food increases, finding ways to reduce food waste and increase efficiency in food production become increasingly important. Using FFs in livestock feed represent an effective solution, and could provide a sustainable source of nutrition for animals while reducing waste and helping to feed a growing population.

To sum up, the recent findings endorse the use of FFs in animal feed as a strategy to contribute toward reducing the ecological footprint associated with animal production and food waste management, while promoting sustainable economic growth. However, it must be implemented responsibly and with careful consideration of potential risks and benefits to human health.

## Data Availability

Not applicable. This study does not involve the use or generation of any specific data or materials.
